# The lncRNA MALAT1 acts as a competing endogenous RNA to regulate KRAS expression by sponging miR-217 in pancreatic ductal adenocarcinoma

**DOI:** 10.1038/s41598-017-05274-4

**Published:** 2017-07-12

**Authors:** Pingping Liu, Haiyan Yang, Jing Zhang, Xiaozhong Peng, Zhaohui Lu, Weimin Tong, Jie Chen

**Affiliations:** 1 Department of Pathology, Peking Union Medical College Hospital, Chinese Academy of Medical Sciences and Peking Union Medical College, Beijing, 100730 China; 2 The State Key Laboratory of Medical Molecular Biology, Institute of Basic Medical Sciences and School of Basic Medicine, Chinese Academy of Medical Sciences and Peking Union Medical College, Beijing, 100005 China; 3 Department of Pathology, Institute of Basic Medical Sciences and School of Basic Medicine, Chinese Academy of Medical Sciences and Peking Union Medical College, Beijing, 100005 China

## Abstract

The long noncoding RNA (lncRNA) metastasis-associated lung adenocarcinoma transcript-1 (MALAT1) has been shown to play an important role in tumourigenesis. The aim of this study was to investigate the role of MALAT1 in pancreatic ductal adenocarcinoma. MALAT1 is expressed at higher levels in pancreatic ductal adenocarcinoma (PDAC) tissues than in nontumour tissues and in metastatic PDAC than in localized tumours. Patients with PDAC and high MALAT1 expression levels have shorter overall survival than patients with PDAC and low MALAT1 expression levels. Knocking down MALAT1 reduces pancreatic tumour cell growth and proliferation both *in vitro* and *in vivo*. Moreover, MALAT1 knockdown inhibits cell cycle progression and impairs tumour cell migration and invasion. We found that miR-217 can bind MALAT1 and regulate its expression in PDAC cell lines. We also found MALAT1 knockdown attenuates the protein expression of KRAS, a known target of miR-217. After MALAT1 knockdown, KRAS protein expression levels can be rescued through inhibition of miR-217 expression. More importantly, MALAT1 knockdown does not directly affect cellular miR-217 expression but decreases the miR-217 nucleus/cytoplasm ratio, suggesting that MALAT1 inhibits the translocation of miR-217 from the nucleus to the cytoplasm.

## Introduction

Pancreatic ductal adenocarcinoma (PDAC) is one of the most commonly diagnosed cancers worldwide. Specifically, PDAC is the sixth-leading cause of cancer-related death in China and the fourth-leading cause of cancer-related death in the USA. The disease is associated with a median survival time of less than 6 months^1, 2^. Surgical resection remains the only potentially curative treatment for pancreatic cancer, but most patients with PDAC present with advanced and/or metastatic disease. Moreover, pancreatic cancer is largely resistant to radiotherapy and chemotherapy, and little progress has been made with respect to its treatment in past decades. Advances in the understanding of PDAC biology may provide clinicians with new tools that can be used for the treatment of this disease.

Noncoding RNAs (ncRNAs) include microRNAs (miRNAs) and long noncoding RNAs (lncRNAs)^3, 4^. MiRNAs are short (typically 18–23 nucleotides) single-stranded RNAs that cause posttranscriptional gene silencing by inducing mRNA degradation or repressing translation upon binding to the 3′-untranslated region (UTR) of their target mRNAs^5^. This process is modulated by the RNA-induced silencing complex (RISC), which contains argonaute2 (Ago2) as its catalytic component^6^. LncRNAs are transcripts >200 nucleotides in length that have also been shown to regulate gene expression^7^. Popular opinion suggests that lncRNAs form extensive networks of ribonucleoprotein complexes to regulate gene expression^8^. lncRNAs also pair with miRNAs and titrate them away from their mRNA targets^9^. Increasing amounts of evidence indicate that interactions between miRNAs and lncRNAs play important roles in gene expression regulation, both under physiologic conditions and in cancer.

However, evidence regarding the roles of lncRNAs and miRNAs in PDAC remains limited. It has been reported that the lncRNAs HOTAIR and LOC389641 are upregulated in PDAC and exhibit pro-oncogenic activity^10, 11^. We previously found that MIR31HG is markedly upregulated and negatively regulated by miR-193b in PDAC^12^. Initial data-mining studies of publically available databases^13–15^ showed that three lncRNAs, namely, metastasis-associated lung adenocarcinoma transcript-1 (MALAT1), LOC100190986 and small nucleolar RNA host gene 12 (SNHG12), were expressed at higher levels in PDAC tissues than in normal pancreas tissues and in metastatic tumours than in localized tumours. Furthermore, all three lncRNAs have been shown to be negative prognostic factors in PDAC. MALAT1, also known as nuclear-enriched abundant transcript-2 (NEAT2), is a highly abundant and conserved nuclear lncRNA^16^ that was originally identified as a prognostic marker for the survival of patients with stage I lung cancer^17^ but has since been shown to play an important role in the carcinogenesis and metastasis of various cancers, including liver, prostate, colorectal and gallbladder cancer^18–21^. Interestingly, evidence exists indicating that miRNAs may regulate MALAT1 expression and function. MALAT1 has been shown to have binding sites for miR-125b, which can downregulate MALAT1 expression and inhibit bladder cancer development^22^. It has also been reported that MALAT1 functions as a competing endogenous RNA to regulate Rac1 expression by sequestering miR-101b in liver fibrosis^23^. Researchers recently uncovered evidence indicating that MALAT1 promotes osteosarcoma development by targeting TGFA via miR-376A^24^. However, little is known about the interaction between miRNAs and MALAT1 in PDAC. MiR-217 has been reported to be a potential tumour suppressor in osteosarcoma, breast cancer, gastric cancer, colorectal cancer, lung cancer and oesophageal cancer^25–29^. Furthermore, we have determined that miR-217 is downregulated in PDAC and functions as a tumour suppressor gene by inhibiting KRAS translation^30^. Bioinformatics analyses have revealed that three putative binding sites for miR-217 can be found in the MALAT1 sequence (NR_002819.2), suggesting that this miRNA can regulate MALAT1 expression and function. However, whether an interaction involving miR-217, MALAT1 and KRAS occurs in pancreatic cancer remains unknown. The present study was designed to examine MALAT1 expression and function in pancreatic cancer, to determine whether MALAT1 is a target of miR-217 and to elucidate the interaction between MALAT1 and KRAS. The main findings of the study were as follows: MALAT1 promotes oncogenesis and enhances KRAS/mitogen-activated protein kinase (MAPK) signalling pathway activation in pancreatic cancer at least in part through a direct interaction with miR-217.

## Results

### MALAT1 is highly expressed in PDAC, and its expression is inversely related to survival

**Figure 1:**
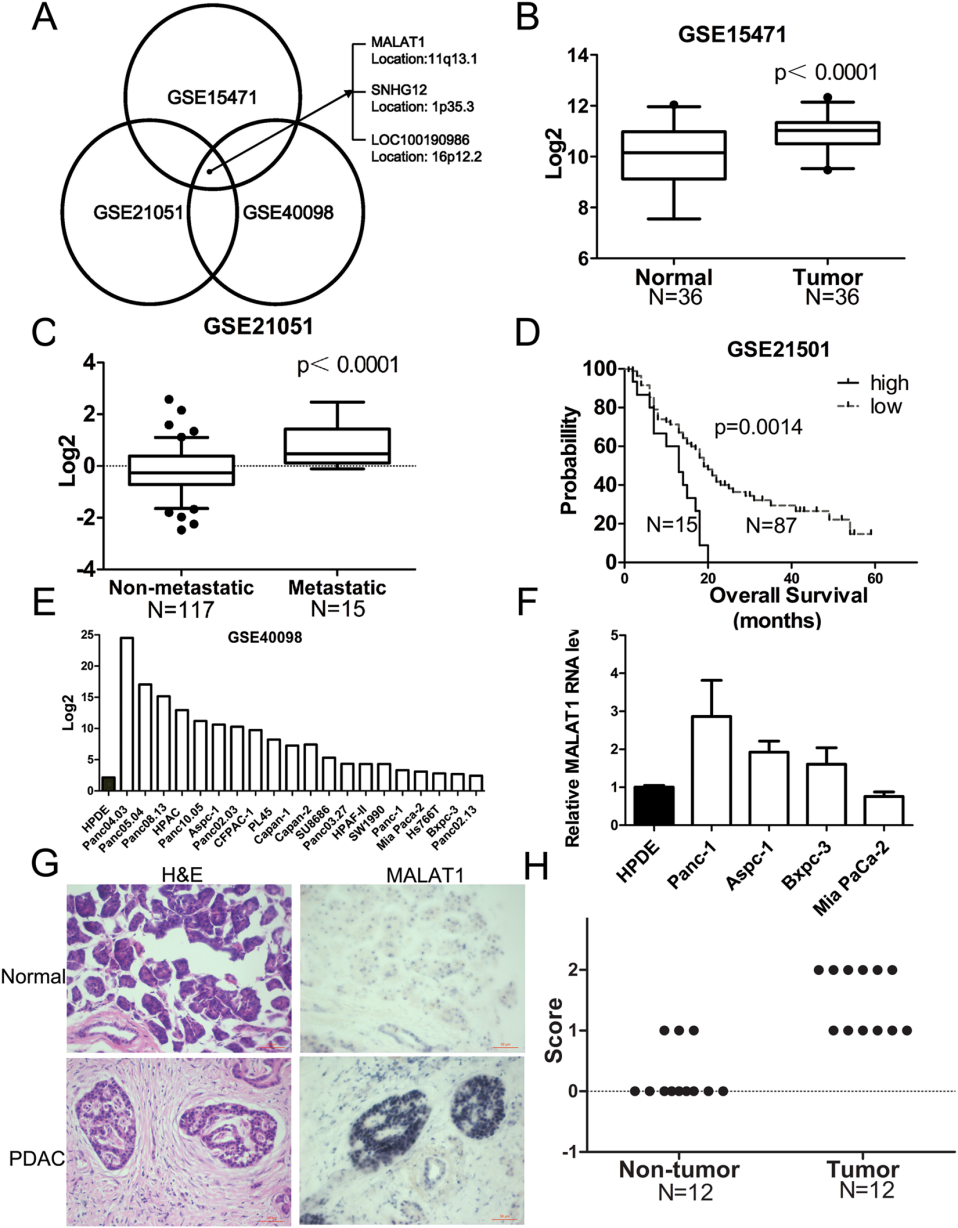
MALAT1 expression and prognostic significance in pancreatic cancer. (**A**) LncRNAs that were overexpressed in all three available sets of gene profiling results and were associated with survival. (**B**) MALAT1 expression in normal pancreatic tissues and pancreatic tumour tissues from patients. (**C**) Relative expression levels of MALAT1 in patients with localized pancreatic tumours and patients with distant metastasis. (**D**) Kaplan–Meier plot showing the survival of patients with pancreatic cancer, according to whether such patients expressed high and low levels of MALAT1. (**E**) MALAT1 expression in pancreatic cancer cells, according to the array data. (**F**) MALAT1 expression in pancreatic cancer cells, as determined by reverse transcription and quantitative real-time PCR. (**G**) MALAT1 expression in normal pancreatic tissues and pancreatic tumour tissues from patients, as determined by *in situ* hybridization. H&E: Haematoxylin and eosin staining. Bar = 50 μm. (**H**) Reaction intensity was scored as undetectable or low (0), moderate (1) or strong (2).

The results of a publically available gene profiling analysis^14^ showed that among 36 patients with pancreatic cancer, 2308 lncRNA probes were markedly upregulated in pancreatic cancer tissue compared with adjacent normal tissue, and 356 lncRNA probes were downregulated in pancreatic cancer tissue compared with adjacent normal tissue (fold change ≥1, p < 0.05). We subsequently analysed the upregulated lncRNAs using another publically available gene expression profile set^13^ and found that 113 lncRNA probes were more highly expressed in metastatic tumours from patients with cancer than in primary tumours from patients with cancer (fold change ≥1.0, p ≤ 0.05). We also verified whether these lncRNAs were also overexpressed in PDAC cell lines^31^. We prioritized lncRNAs that were upregulated in all three publically available gene expression profiles and were related to overall post-surgery patient survival. This filtering process identified the following 3 lncRNAs: MALAT1, LOC100190986 and SNHG12 (Fig. 1A).

Data mining of the above gene profiling results showed that MALAT1 was expressed at higher levels in PDAC tissues than in nontumour tissues (Fig. 1B) and in metastatic PDAC than in localized PDAC (Fig. 1C). Survival analysis (Fig. 1D) revealed that patients with high MALAT1 expression (the upper 15%) had significantly decreased overall survival compared with patients with low MALAT1 expression (the lower 85%). Additional summaries of these patient data are provided in Supplementary Table S1. Data mining of the array data pertaining to several pancreatic cancer cell lines showed that MALAT1 was overexpressed in Aspc-1, Panc-1 and BxPC-3 cells (Fig. 1E). These data were supported by our observation that MALAT1 was highly expressed in Aspc-1, Panc-1 and BxPC-3 cells but lowly expressed in Mia Paca-2 cells compared with immortalized human pancreatic duct (HPDE) cells (Fig. 1F). Figure 1G and H illustrate the results of the RNA *in situ* hybridization experiments and show that MALAT1 was highly expressed in PDAC tissue compared with nontumour tissue. MALAT1 fragments were detectable in human serum; however, their levels could not be used to distinguish PDAC tissues from chronic pancreatitis tissues and healthy control tissues (Fig. S1).

### Silencing MALAT1 impairs cell proliferation and cell cycle progression and promotes cell apoptosis

**Figure 2:**
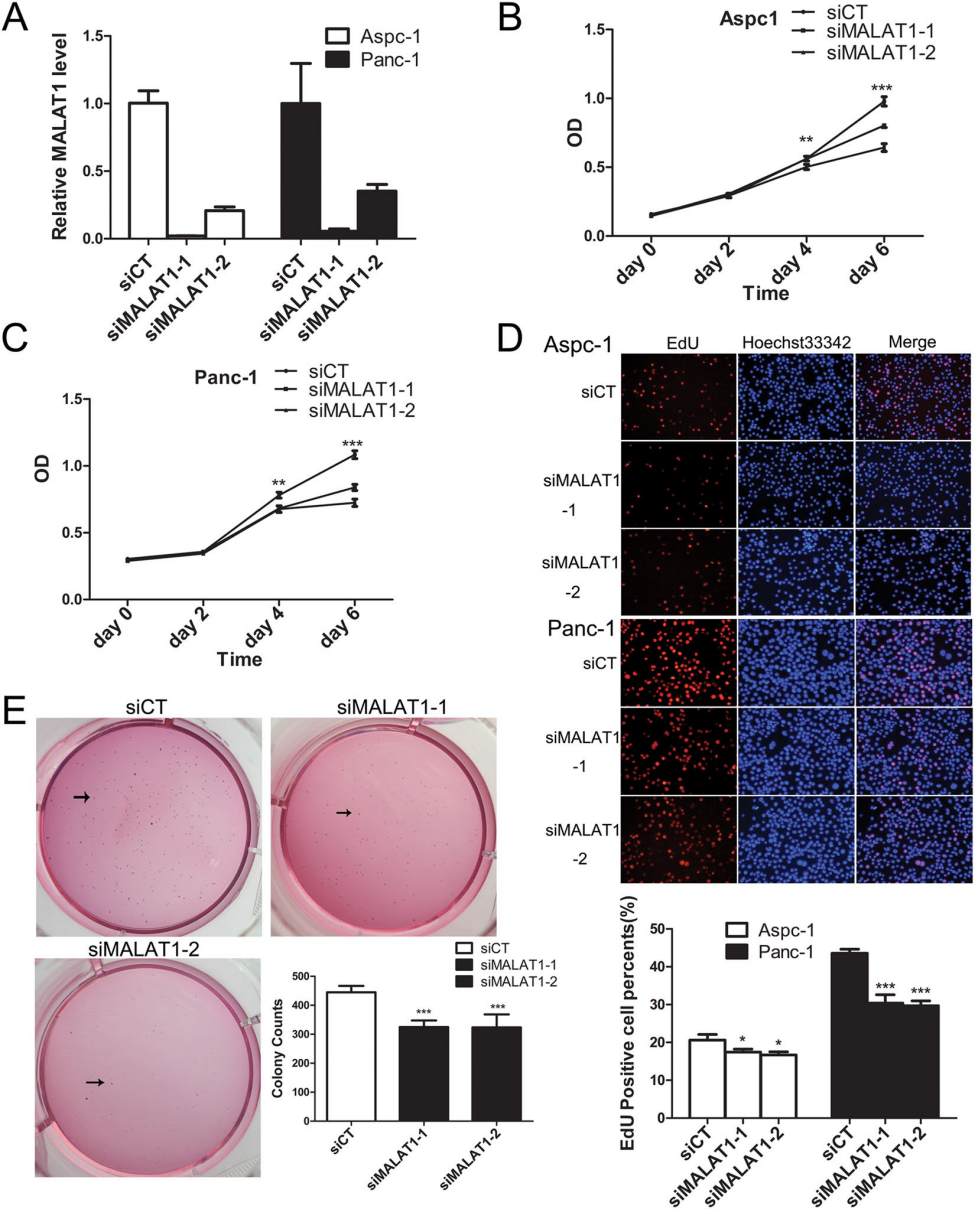
MALAT1 knockdown inhibits cell proliferation. (**A**) MALAT1 mRNA expression after siRNA-mediated MALAT1 knockdown in pancreatic cancer cells (siCT: negative control, siMALAT1-1 and siMALAT1-2). (**B**) Cell growth in Aspc-1 and Panc1 cells, as determined by CCK8 assay (**C**) transfected with siRNAs. (**D**) Cell proliferation in Aspc-1 and Panc1 cells transfected with siRNAs, as determined by EdU assay. (**E**) Anchorage-independent growth of PDAC cells after MALAT1 downregulation. All the quantitative results in this study are presented as the mean ± standard error of the mean of at least three replicate determinations of each data point. *p < 0.05, **p < 0.01, ***p < 0.001 for siMALAT1 vs siCT.

**Figure 3:**
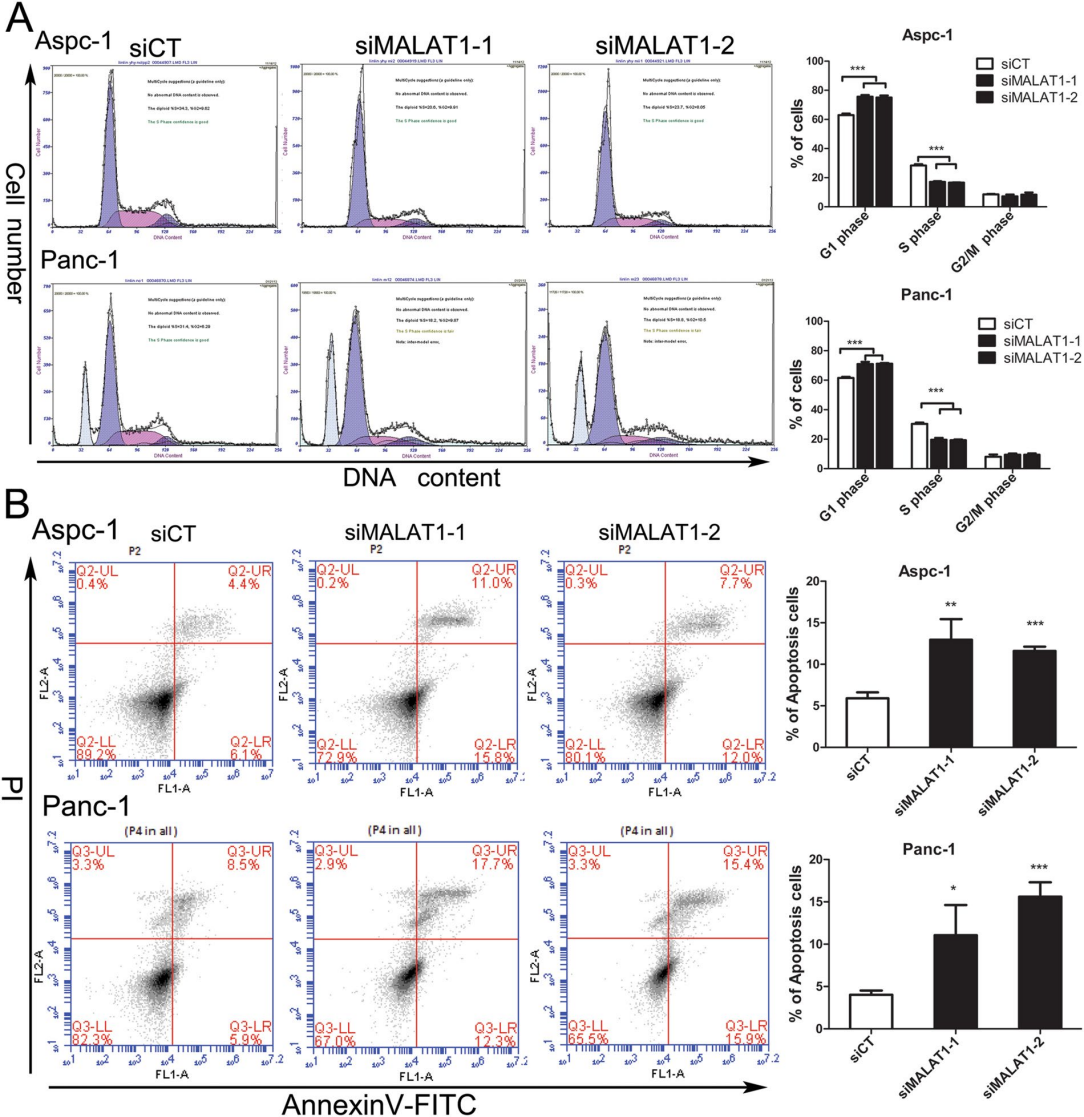
MALAT1 knockdown inhibits cell cycle progression and promotes cell apoptosis. (**A**) Effects of MALAT1 knockdown on cell cycle progression in pancreatic cancer cells (PI staining). (**B**) Effects of MALAT1 knockdown on cell apoptosis in pancreatic cancer cells (Annexin V-FITC staining). All the quantitative results in this study are presented as the mean ± standard error of the mean of at least three replicate determinations of each data point. *p < 0.05, ***p < 0.001 for siMALAT1 vs siCT.

Gene ontology analysis of the above study data indicated that MALAT1 regulates gene sets associated mainly with cell growth and proliferation, cell apoptosis and cell death, cellular localization and cellular metabolic processes (Fig. S2). To verify the role of MALAT1 in pancreatic tumours, we knocked down MALAT1 in Panc-1 and Aspc-1 cells using two siRNAs (siMALAT1-1 and siMALAT1-2) to avoid off-target effects. The two above mentioned cell lines were chosen for this study because both express relatively high levels of MALAT1 compared with HPDE cells. MALAT1 knockdown efficiency was determined by qPCR. MALAT1 knockdown resulted in a 64.3–97.8% reduction in MALAT1 RNA expression in PDAC cells (Fig. 2A). CCK-8 and EdU assays were used to examine PDAC cell growth. CCK-8 proliferation assay showed that MALAT1-knockdown Aspc-1 and Panc-1 cells exhibited decreased cell growth compared with control cells (Fig. 2B and C), and EdU labelling assay confirmed that cell proliferation was reduced in MALAT1-knockdown cells compared with control cells (Fig. 2D). Furthermore, MALAT1 affected tumour cell anchorage-independent growth, and cells transfected with siMALAT1-1 or siMALAT1-2 siRNA displayed fewer and smaller colonies in soft agar than control cells (Fig. 2E). To elucidate the mechanism underlying MALAT1-knockdown mediated proliferation inhibition, we conducted cell cycle progression and cell apoptosis assays in PDAC cells. Cell cycle analysis revealed that MALAT1 knockdown induced cell cycle arrest in G1 phase, an effect accompanied by a significant increase in the size of the G1 cell population and a decrease in the size of the S-phase cell population in both Panc-1 and Aspc-1 cells (Fig. 3A). Annexin V staining revealed that MALAT1 knockdown increased the percentage of early apoptotic cells among treated cells compared with control cells (Fig. 3B). Taken together, these results indicate that MALAT1 enhances pancreatic cancer cell growth and proliferation and inhibits cell apoptosis.

### Silencing MALAT1 inhibits tumour cell migration, invasion and growth *in vivo*

**Figure 4:**
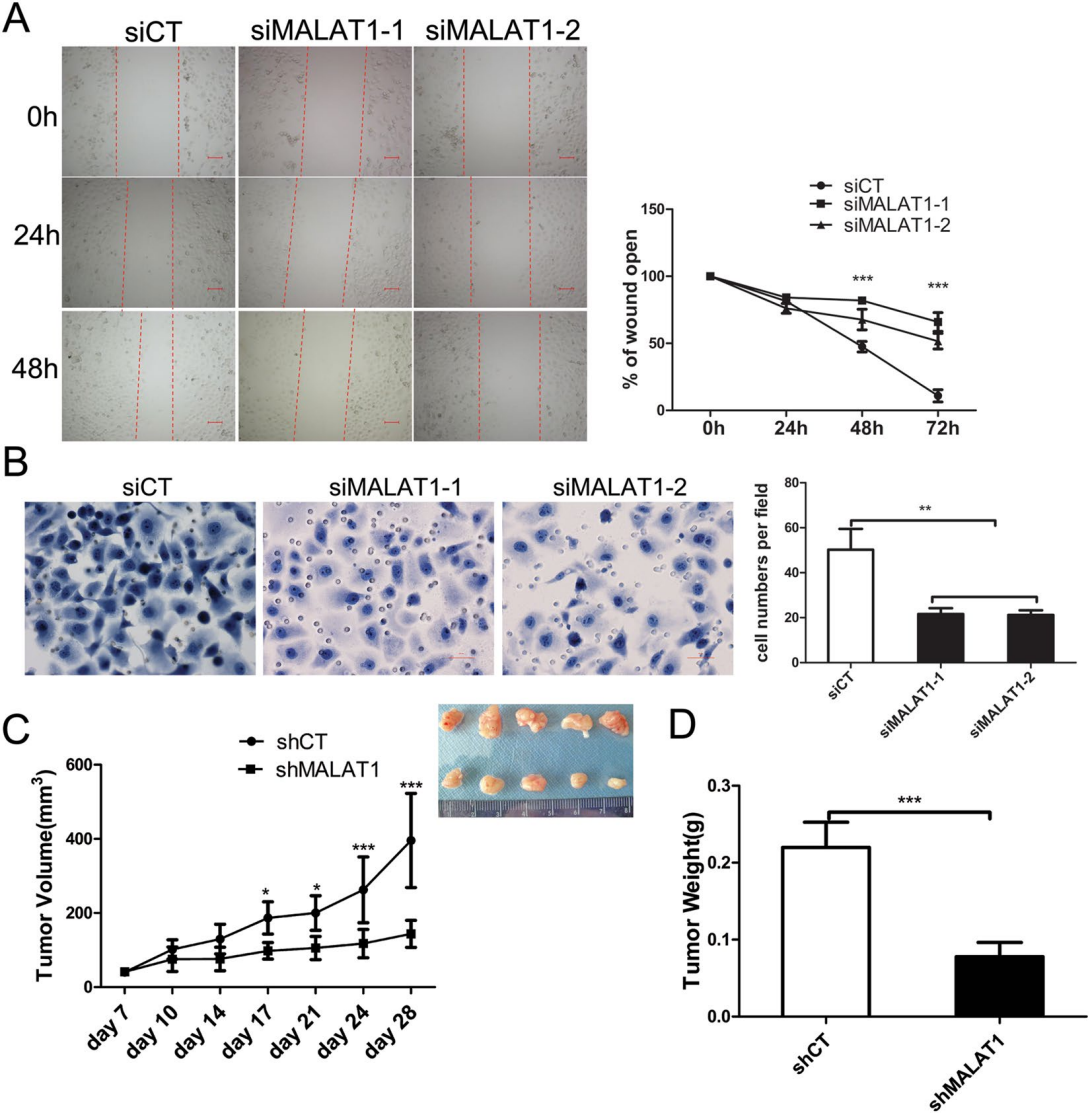
MALAT1 knockdown inhibits tumour cell migration, invasion and growth *in vivo*. (**A**) Closure of wounds made in a confluent monolayer of Panc-1 cells transfected with siRNAs. For each experimental group, the percentage of the wound remaining open, which was determined based on the size of the wound at 0 hours, is shown. (**B**) The effects of MALAT1 knockdown on cell invasion, as determined by the transwell invasion assay. (**C**) Panc-1 cells transfected with shCT vector or shMALAT1 vector were subcutaneously injected into the posterior flanks of nude mice. Tumour volumes were measured at regular intervals. Finally, the tumours were photographed and weighed (**D**). All the quantitative results in this study are presented as the mean ± standard error of the mean of at least three replicate determinations of each data point. *p < 0.05, **p < 0.01, ***p < 0.001 for siMALAT1 vs siCT.

To investigate whether MALAT1 is associated with pancreatic cancer progression further, we analysed the effects of MALAT1 knockdown on pancreatic cancer cell migration and invasion. Knocking down MALAT1 in Panc-1 cells significantly inhibited cell migration during the closure of an artificial wound created over a confluent cell monolayer (Fig. 4A). In addition, knocking down MALAT1 dramatically inhibited the normally strong invasive capacity of Panc-1 cells (Fig. 4B).

The above findings were verified in an *in vivo* model. Panc-1 cells transfected with shCT or shMALAT1 vector were subcutaneously injected into the posterior flanks of nude mice. After 4 weeks, we found that tumour growth was significantly slower in mice transfected with the shMALAT1 vector than in those transfected with the shCT vector (Fig. 4C). Consistent with the tumour growth curve results, the results regarding tumour weights showed that the mean weight of the tumour induced by the shMALAT1 vector was significantly lower than that induced by the shCT vector on day 28 post-injection (Fig. 4D).

### MiR-217 regulates MALAT1 expression

**Figure 5:**
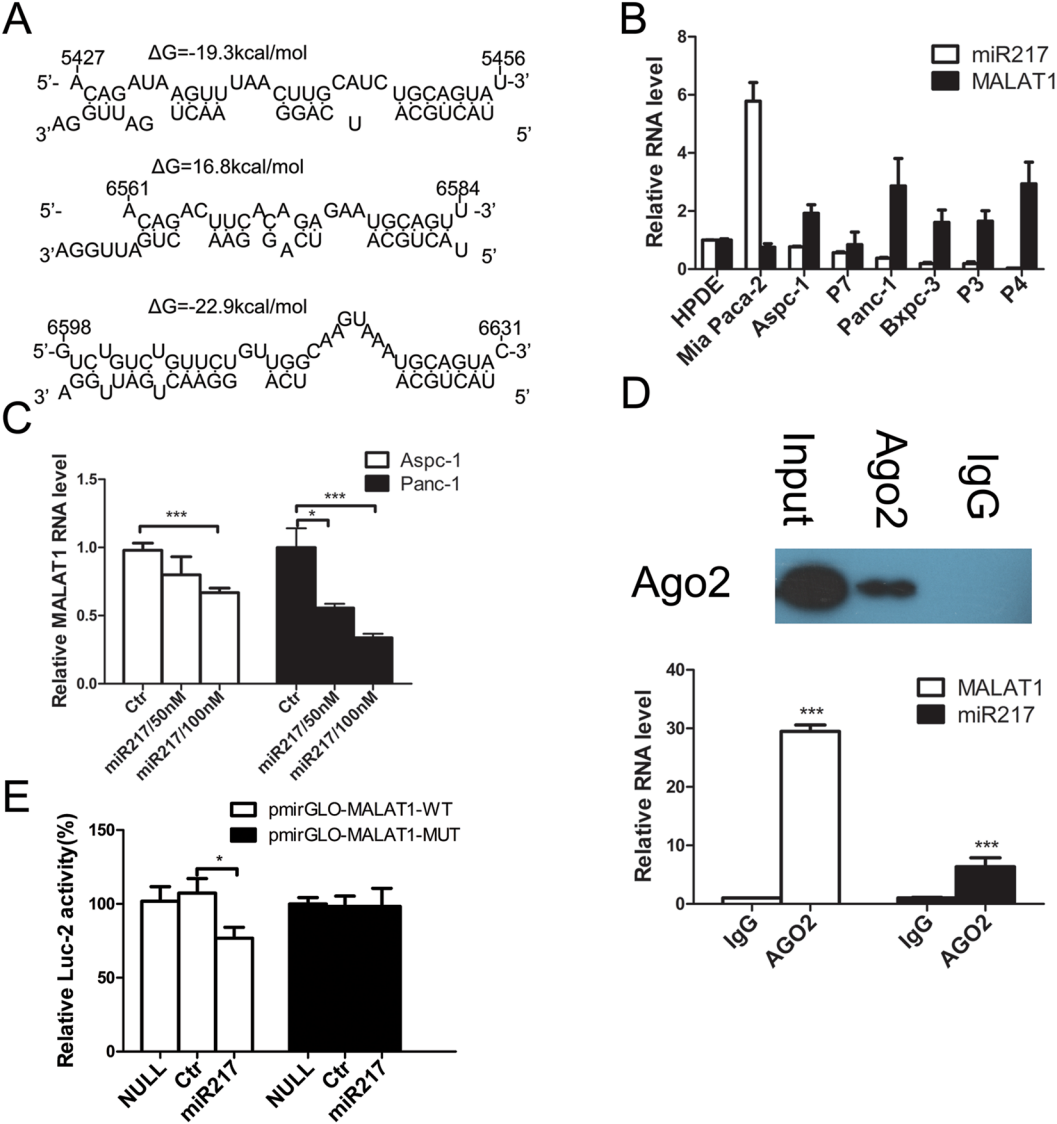
MALAT1 is negatively regulated by miR-217 in pancreatic cancer. (**A**) Bioinformatics analysis predicted that miR-217 binding sites were present at three distinct positions in MALAT1. The nucleotides are numbered based on their positons relative to the MALAT1 transcriptional start site. (**B**) The relative expression levels of MALAT1 and miR-217 in various pancreatic cancer cells. (**C**) MALAT1 expression in pancreatic cancer cells after transfection with an RNA mimic of miR-217. (**D**) Coimmunoprecipitation with mouse monoclonal anti-Ago2 (Ago2) or preimmune IgG from extracts of Panc-1 cells. Upper panel: Immunoprecipitation using Ago2 or IgG, followed by western blot analysis using a rabbit monoclonal anti-Ago2 antibody. Lower panel: mRNA levels in immunoprecipitates, as determined by RT-qPCR. (**E**) Luciferase reporter gene expression after pmirGLO-MALAT1-WT vectors or pmirGLO-MALAT1-MUT vectors were transfected into PDAC cells along with miR-217 or negative-control RNA.

Bioinformatics algorithms used for predicting miRNA targets, including miRcode^32^ and RNAhybrid^33^, identified miR-217 as one of the miRNAs specific for MALAT1. Analysis of the alignment of miR-217 with MALAT1 sequences from different species showed that the MALAT1 sequence was relatively well conserved between humans and primates. The sites at positions 5,245 and 6,561 (Fig. 5A) may be considered well and moderately conserved, with average conservation scores of 0.78 and 0.67, respectively. The site at position 6,598 (Fig. 5A) is less well conserved than the above two sites, with an average conservation score of 0.56. Taken together, these results suggest that MALAT1 is potentially a direct target of miR-217.

MiR-217 and MALAT1 expression levels in a panel comprising seven human PDAC cell lines (Panc-1, MIA Paca-2, Aspc-1, Bxpc-3, P3, P4 and P7) and HPDE cells were assessed by qPCR (Fig. 5B). The results of this assessment suggested that a negative correlation exists between miR-217 and MALAT1. Similar results were noted in the experiments pertaining to the clinical specimens. For example, the results of the *in situ* hybridization study showed that MALAT1 expression was upregulated (Fig. 1G), whereas miR-217 expression was downregulated in PDAC tissues compared with adjacent nontumour tissues^30^. Both MALAT1 and miR-217 were localized predominantly in the nuclei of the ductal epithelial cells of the PDAC and nontumour tissue samples^30^. These findings suggest that MALAT1 may be directly regulated by miR-217 in PDAC.

To determine whether miR-217 regulates MALAT1, we transfected 50 or 100 nM RNA mimics of miR-217 RNA into Aspc-1 and Panc-1 cells. The results of these experiments showed that miR-217 inhibited MALAT1 RNA expression in a concentration-dependent manner (Fig. 5C). To determine whether MALAT1 associates with RISCs, we performed coimmunoprecipitation experiments in extracts of Panc-1 cells using antibodies against Ago2. We found that MALAT1 was preferentially enriched (29.5 fold) in Ago2-containing miRISCs compared with control immunoglobulin G (IgG) immunoprecipitates (Fig. 5D, lower panel, left column), while miR-217 was associated with RISCs (Fig. 5D, lower panel, right column). The specificity of the Ago2 antibody was confirmed by immunoprecipitation and immunoblotting analyses (Fig. 5D, upper panel). Thus, MALAT1 is present in Ago2-containing RISCs, likely as a result of its association with miR-217 or other miRNAs.

To confirm that MALAT1 is a direct target of miR-217, we performed experiments using pmirGLO-MALAT-wild-type (WT) to determine the functionality of the three predicted miR-217 binding sites. As expected, the cotransfected miR-217 RNA mimic inhibited Firefly luciferase expression (Fig. 5E, left column). To confirm that this inhibition was dependent on the predicted miR-217 sites, we tested a pmirGLO-MALAT-MUT whose recognition sites contained mutations (Fig. S3, the substituted nucleotides are underlined). This mutant no longer responded to the miR-217 RNA mimic (Fig. 5E, right column). Taken together, these results suggest that the three predicted miR-217 binding sites contain functional miR-217 binding sites.

### MALAT1 knockdown reduces KRAS expression

**Figure 6:**
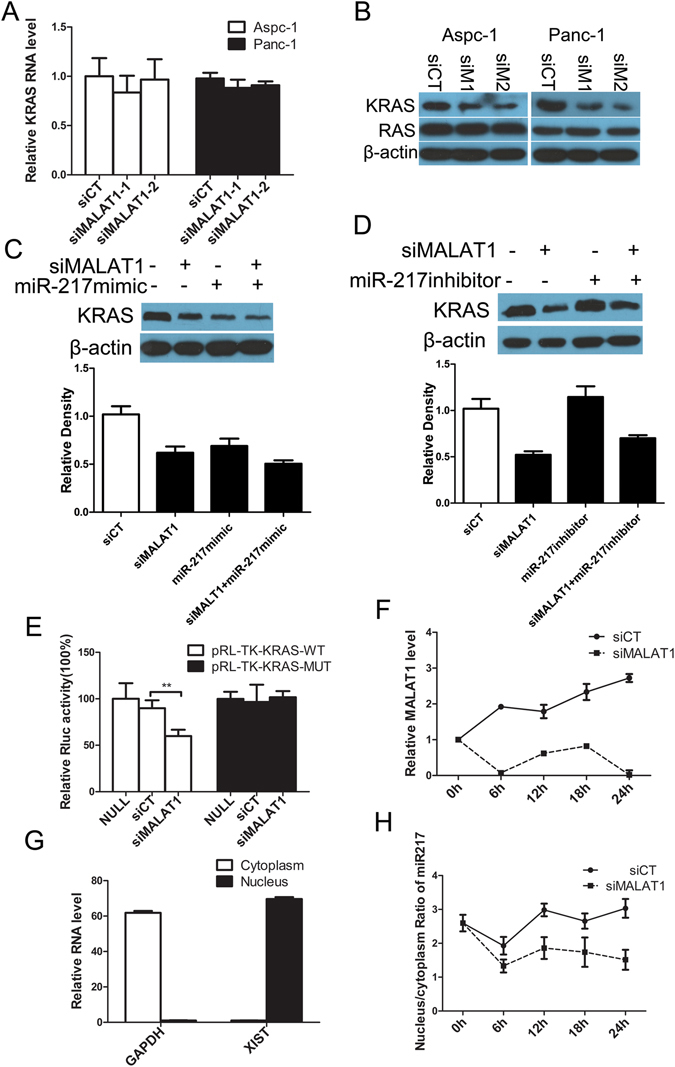
MALAT1 regulates KRAS expression in pancreatic cancer cells by interacting with miR-217. (**A**) KRAS mRNA expression after MALAT1 knockdown in pancreatic cancer cells. (**B**) KRAS and RAS protein expression after MALAT1 knockdown in pancreatic cancer cells. β-actin was used as a loading control. siCT: negative control siRNA, siM1: siMALAT1-1, siM2: siMALAT1-2. (**C**) KRAS protein expression in pancreatic cancer cells after the cells were transfected with negative-control siRNA (siCT) or siMALAT1 with or without an miR-217 mimic or miR-217 inhibitor (**D**). The full-length gels and blots for KRAS protein are included in Fig. S5. (**E)** Luciferase reporter gene expression after pRL-TK-KRAS-WT vectors or pRL-TK-KRAS-MUT vectors were transfected into pancreatic cancer cells along with either siMALAT1 or negative-control siRNA (siCT). (**F**) MALAT1 downregulation after pancreatic cancer cells were transfected with siRNAs. (**G**) XIST and GAPDH RNA levels in the nuclear and cytoplasmic fractions, as determined by qPCR. (**H**) miR-217 nucleus/cytoplasm ratios in pancreatic cancer cells after the cells were transfected with siCT or siMALAT1.

Since both MALAT1 and KRAS are targets of miR-217^30^, we investigated whether MALAT1 affects KRAS expression by binding miR-217. We observed a selective decrease in KRAS protein levels, other than total RAS protein levels, after knocking down MALAT1 (Fig. 6B), while KRAS mRNA levels were unchanged (Fig. 6A). MiR-217-mediated translational repression of KRAS without RNA destabilization has been reported previously^30^. Taken together, these results suggest that MALAT1 knockdown and miR-217 overexpression have similar effects on KRAS expression. Moreover, the combination of miR-217 overexpression and MALAT1 knockdown resulted in a larger reduction in KRAS protein levels than either method alone (Fig. 6C). Additionally, the decreases in KRAS levels resulting from MALAT1 knockdown could be partially rescued by the introduction of an miR-217 inhibitor (Fig. 6D).

To determine whether MALAT1 regulates KRAS protein expression through miR-217 binding, we used a pGL3 control plasmid to transfect a pRL-TK-KRAS-WT vector containing two miR-217 recognition sites and a pRL-TK-KRAS-MUT vector whose recognition sites contained mutations into Aspc-1 and Panc-1 cells along with either siRNA. The results of the luciferase assays indicated that Renilla luciferase expression was downregulated in the presence of siMALAT1-1 in the pRL-TK-KRAS-WT panel (Fig. 6E, left column), indicating that MALAT1 binds miR-217 to act as a decoy and to abolish the repressive effects of miR-217 on KRAS. This effect was negated when pRL-TK-KRAS-MUT was used (Fig. 6E, right column). Taken together, these results suggest that MALAT1 inhibits endogenous miR-217 function, leading to derepression of KRAS RNA translation.

KRAS gene mutations, which occur in more than 90% of pancreatic intraepithelial neoplasms and carcinomas, are a major cancer initiating event and are required for pancreatic carcinogenesis and tumour progression^34^. These mutations compromise the ability of the Ras protein to hydrolize GTP to GDP, effectively locking the protein in an active conformation, leading to stimulation of the Ras/Raf/MAPK pathway^34, 35^. Therefore, we employed western blotting to detect the activation levels of the MAPK pathway following MALAT1 knockdown in Panc-1 cells. Our data demonstrated that MALAT1 knockdown induced a significant decrease in MEK and ERK phosphorylation levels in knockdown cells compared with negative-control cells (Fig. S4). However, no significant differences in total MEK and ERK levels were observed in the former cells compared with the latter cells.

**Figure 7:**
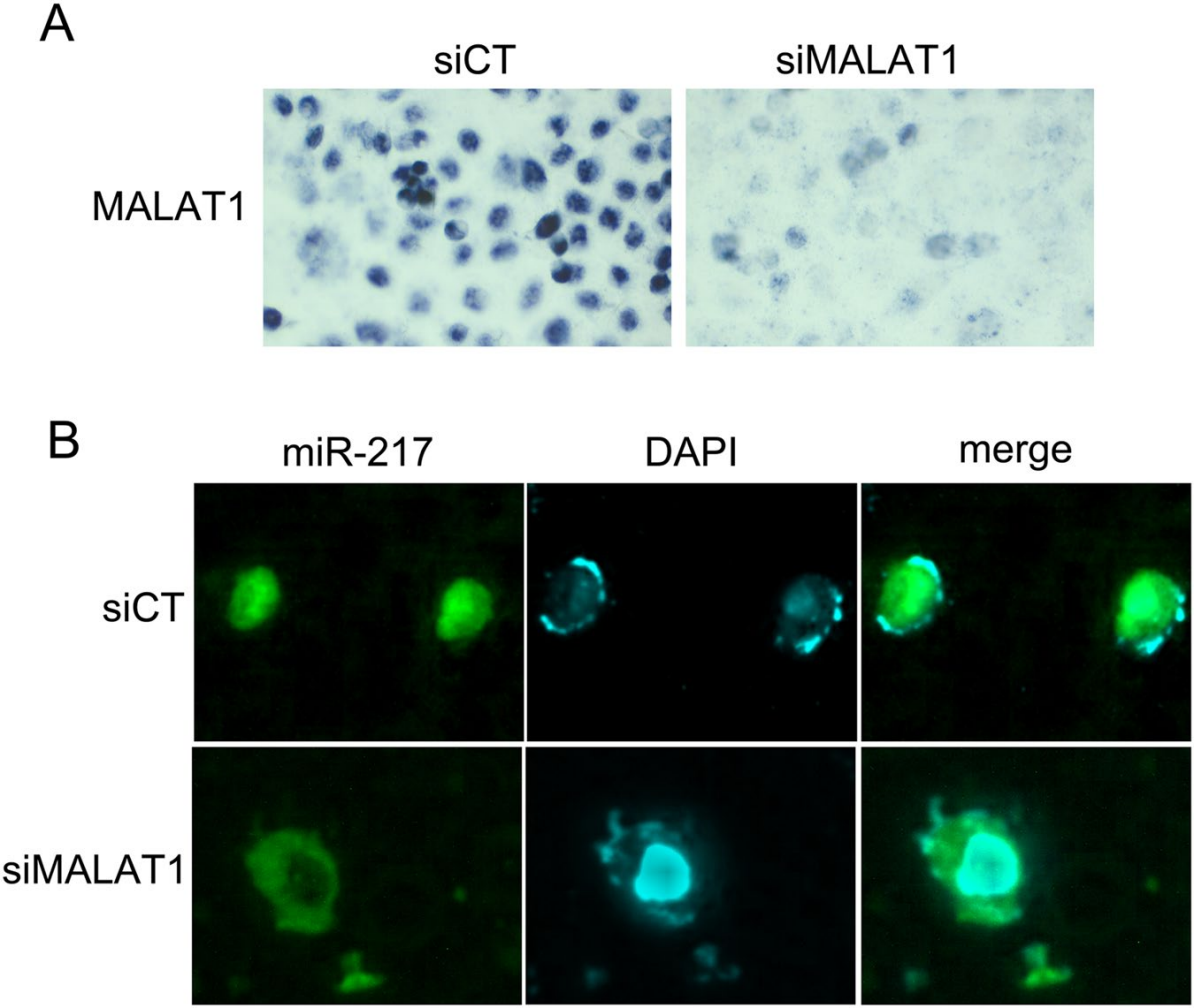
MALAT1 kncokdown (A) promoted the migration of miR-217 from the nucleus to the cytoplasm (B).

MiRNAs are known to exert their functions mainly in the cytoplasm; however, MALAT1 is a lncRNA that localizes in nuclear speckles, which raises the question as to how MALAT1 regulates KRAS protein expression via miR-217. *In situ* hybridization has shown that miR-217 is expressed both in the nucleus and in the cytoplasm but is predominantly expressed in the nucleus^30^. Furthermore, miR-217 overexpression downregulates MALAT1 (Fig. 5C), while MALAT1 knockdown has no effect on total miR-217 levels (Fig. S5). We subsequently assessed whether MALAT1 downregulation altered the spatial distribution of miR-217, i.e., whether MALAT1 downregulation promoted the migration of miR-217 from the nucleus to the cytoplasm to repress KRAS mRNA translation. Thus, we transfected Panc-1 and Aspc-1 cells with the siMALAT1 oligonucleotide or control oligonucleotide (Fig. 6F), isolated the nuclear and cytoplasmic fractions, and undertook analyses of the distribution of miR-217 in the subcellular compartments of PDAC cells. Successful isolation of pure nuclear and cytoplasmic fractions was confirmed by qPCR using nuclear-specific RNA X inactivation-specific transcript (XIST) and cytoplasm-specific glyceraldehyde-3-phosphate dehydrogenase (GAPDH) RNA. XIST displayed 69.6-fold enrichment in the nucleus, and GAPDH displayed 61.8-fold enrichment in the cytoplasm (Fig. 6G). Cytoplasmic miR-217 levels increased in response to MALAT1 downregulation, accompanied by a corresponding decrease in nuclear miR-217 levels (a decrease in the nuclear/cytoplasmic ratio), findings suggestive of the translocation of miR-217 from the nucleus to the cytoplasm (Fig. 6H). Then we exzamined the distribution change of miR-217 by fluorescence RNA *in situ* hybridization after knocking down MALAT1. MiR-217 migrates from the nucleus to the cytoplasm after MALAT1 downregulation (Fig. 7A,B).

## Discussion

It has been reported that mRNAs, transcribed pseudogenes, circular RNAs and other lncRNAs can bind miRNA and act as potent miRNA sponges^36–39^. Consistent with these findings, data from recent studies suggest that coding and noncoding RNAs can regulate each other through their ability to competitively bind miRNA. These molecules have been termed competing endogenous RNAs (ceRNAs)^9^. CeRNAs can sequester miRNAs, thereby protecting their target RNAs from repression^36, 40^. In this study, we showed that nuclear MALAT1 lncRNA acts as a competing endogenous RNA to sequester miR-217 in the nucleus and regulate its target gene, KRAS, in a molecular circuit. The significance of this interaction is underscored by our observation that MALAT1 knockdown has a similar effect to miR-217 overexpression on pancreatic carcinogenesis, decreases KRAS protein levels and downregulates the MAPK signalling pathway.

Several studies have reported that MALAT1 displays increased expression in a variety of tumours^17–21, 41^, In this study, we mined publically available gene profiling data and found that MALAT1 is upregulated in pancreatic tumours and PDAC cell lines and is expressed at higher levels in primary tumours from patients with distant metastasis than in localized tumours. We confirmed that MALAT1 is upregulated in pancreatic tumours and PDAC cell lines. These findings are supported by those of studies from other laboratories showing that MALAT1 is highly expressed in pancreatic cancer tissues compared with adjacent non-cancerous tissues; MALAT1 expression levels are positively correlated with clinical stages, lymph node metastases and distant metastases; and MALAT1 expression is an unfavourable prognostic factor in patients with pancreatic cancer^41, 42^. Plasma levels of MALAT1-derived fragments are significantly elevated in patients with prostate cancer compared with those without prostate cancer^43^. However, we observed that the levels of these fragments were not significantly different between healthy controls and patients with pancreatic tumours or chronic pancreatitis. These results suggest that the plasma levels of MALAT1-derived fragments are not a useful biomarker for the diagnosis and prognosis of pancreatic cancer.

Recent studies have shown that lncRNAs can be direct targets for miRNAs^44^. Therefore, we investigated whether MALAT1 is a target for miRNAs in pancreatic tumours. Bioinformatics analysis revealed the presence of many predicted binding sites for miRNAs within the MALAT1 sequence. The results of the qPCR experiments indicated that of the miRNAs predicted to bind the MALAT1 sequence, only miR-217 was able to repress MALAT1 RNA expression in PDAC cells. These results were supported by the results of the experiments in which RNA mimics were transfected into PDAC cells. Furthermore, we noted the existence of a negative correlation between MALAT1 and miR-217 expression in pancreatic tumour specimens and cell lines. Coimmunoprecipitation experiments demonstrated that MALAT1 and miR-217 physically interact *in vivo*, and a luciferase assay confirmed that MALAT1 is a direct target of miR-217 in various cell types. In herpesvirus, the lncRNA HSURs has been shown to bind to and cause the degradation of human miR-27, possibly producing a cellular environment permitting viral infection and transformation^45^. Zhang and colleagues demonstrated that miR-21 can target and suppress the lncRNA growth arrest-specific 5 (GAS5) and that GAS5 could also repress miR-21 expression^44^. However, in our study^44^, MALAT1 knockdown had no influence on the levels of miR-217 but still induced a decrease in the levels of KRAS protein, which is also a target for miR-217. Furthermore, luciferase assays showed that MALAT1 knockdown induced endogenous miR-217 to repress pRL-TK-KRAS-WT reporter gene expression. These findings implies that MALAT1 binds and sequesters miR-217 to protect KRAS mRNA from repression but does not degrade miR-217.

According to the ceRNA hypothesis, lncRNAs may elicit their biological effects through their ability to act as endogenous decoys for miRNAs. Such activity would affect the ability of miRNAs to bind their targets^9^. In pancreatic tissue, miR-217 predominantly localizes in the nucleus and displays a punctate staining pattern in the cytoplasm, while MALAT1 and KRAS RNA are expressed in the nucleus and cytoplasm, respectively. We hypothesize that MALAT1 regulates KRAS expression by influencing the spatial distribution of miR-217. Consistent with this finding, we found that the miR-217 nucleus/cytoplasm ratio decreased after MALAT1 knockdown, suggesting that miR-217 had translocated from the nucleus to the cytoplasm. This study demonstrated for the first time that lncRNA regulates mRNA translation by affecting the spatial distributions of miRNAs.

It is well known that the MAPK pathway acts as one of the major downstream effectors of KRAS signalling. Consistent with our observation that KRAS protein expression levels decreased after MALAT1 knockdown was our observation that MALAT1 knockdown also downregulated the MAPK signalling pathway, which is constitutively activated in pancreatic tumours. A previous study uncovered evidence that the MAPK pathway is inactivated in the GBC cell line after MALAT1 knockdown^20^. Taken together, these findings suggest that MALAT1 may activate the MAPK pathway in solid tumours by regulating KRAS. KRAS is an oncogene that plays a key role in the onset of pancreatic cancer and also plays an essential role in invasive pancreatic cancer. Resistance to KRAS inhibition has been observed experimentally in studies regarding pancreatic cancer treatment^34^; thus, targeting MALAT1 may be another way to achieve KRAS/MAPK pathway inactivation.

The present study demonstrated that MALAT1 knockdown significantly inhibited PDAC cell proliferation and metastasis *in vitro* and/or *in vivo*. A different laboratory recently reported similar findings^46^. The mechanisms underlying these phenomena probably involve the induction of G1/M cell cycle arrest and the promotion of cell apoptosis. Evidence exists indicating that MALAT1 promotes cellular proliferation by modulating the expression and/or pre-mRNA processing of cell cycle-regulated transcription factors or by promoting autophagy *in vitro* ^47, 48^. Moreover, Gutschner and colleagues showed that MALAT1 functions as a critical regulator of the metastatic phenotypes of lung cancer cells by actively regulating the expression of various genes, including a set of metastasis-associated genes^49^. The findings of another study indicated that MALAT1 promotes brain metastasis by inducing epithelial-mesenchymal transition in lung cancer^43^. MALAT1 can recruit EZH2 to the E-cadherin promoter to represses E-cadherin expression and promote cell migration and invasion in pancreatic cancer cell lines^50^. In this study, we found that MALAT1 promotes pancreatic cancer cellular proliferation and metastasis at least in part by inducing MAPK pathway overactivation. In addition, we found that MALAT1 knockdown promoted cell apoptosis in pancreatic cancer cells. The results of our gene ontology analysis indicated that MALAT1 regulates the expression of gene sets associated not only with cell growth and proliferation, apoptotic processes and cell death but also with cellular localization and metabolic processes, a topic that merits further study.

In conclusion, the main finding of this study was that MALAT1 acts as a tumour promoter at least in part by binding miR-217 and sequestering the molecule in the nucleus, thereby promoting oncogenic KRAS expression in PDAC.

## Materials and Methods

### Human tissue samples and cell lines

All experiments were conducted in accordance with the relevant guidelines and regulations. This project was approved by the Peking Union Medical College Hospital Clinical Research Ethics Committee. Informed consent was obtained from all subjects prior to their participating in the study and the inclusion of their data in the analysis. Human tissue samples were obtained from patients undergoing surgery for pancreatic cancer in Peking Union Medical College Hospital (China) between January 2013 and December 2013, immediately snap-frozen and stored in liquid nitrogen. The distance between the tumour and para-tumour tissues ranged from 1.5–2.0 cm. Panc-1, Aspc-1, Mia Paca-2 and Bxpc-3 cells were purchased from the American Type Culture Collection (ATCC) and were authenticated by STR profiling between 2012 and 2014. HPDE cells were obtained from the Pancreatic Cancer Lab of the Comprehensive Cancer Therapy Center of the University of Michigan (Ann Arbor, Michigan, United States) in 2013. These cells were authenticated by STR profiling. The P3, P4 and P7 cell lines were constructed in our laboratory in 1990^51^. The Panc-1 and Mia Paca-2 cells were cultured in Dulbecco’s modified Eagle medium (DMEM) supplemented with 10% foetal calf serum (FCS). The Aspc-1, Bxpc-3, P3, P4 and P7 cells were cultured in RPMI-1640 medium, and the HPDE cells were cultured in a complete growth medium containing 75% DMEM without glucose (Sigma), 25% medium M3 base (Incell Corp.), 5% FCS, 10 ng/ml human recombinant epidermal growth factor (EGF), 5.5 mmol/L D-glucose (1 g/L) and 750 ng/ml puromycin. All the cells were cultured at 37 °C in a humidified atmosphere containing 5% CO_2_.

### DNA template and reporter vector construction

To construct a plasmid to serve as a DNA template from which an RNA labelling probe can be transcribed, we amplified a 985-bp MALAT1 DNA fragment using MALAT1 forward and reverse PCR primers. The primers used in this study are listed in Supplementary Table S2. This fragment was inserted into a pGEM-3zf(+) vector between the HindIII and EcoRI sites. To construct reporter vectors, we amplified a MALAT1 DNA fragment containing three WT miR-217 recognition sites from Panc-1 genomic DNA and cloned the fragment into a pmirGLO Dual luciferase miRNA target expression vector (Promega Corporation) immediately downstream of the Firefly luciferase gene and between the XbaI and SacI sites (pmirGLO-MALAT1-WT). Then, we constructed a pmirGLO reporter vector containing a MALAT1 DNA fragment featuring mutations in each of the three seed recognition sites (MUT) between the XbaI and SacI sites (pmirGLO-MALAT1-MUT). pRL-TK-KRAS-WT, which contained two miR-217 recognition sites, was subcloned into a pRL-TK vector (Promega Corporation) downstream of the Renilla luciferase gene, while pRL-TK-KRAS-MUT, whose recognition sites contained mutations, was subcloned into a pRL-TK vector^30^. A pGL3 control plasmid expressing Firefly luciferase was used to monitor transfection efficiency (Promega Corporation).

### Cell fractionation and RNA and protein quantification

Total RNA was isolated using Trizol (Invitrogen), according to the manufacturer’s protocol, and the nuclear and cytoplasmic fractions were prepared according to the manufacturer’s instructions (Promega Corporation). Real-time reverse-transcription PCR assay was performed with SYBR Green PCR Master Mix using a StepOnePlus Detection System (ABI) to detect MALAT1, KRAS, miR-217 and GAPDH mRNA expression levels. All the primers used in this study are listed in Supplementary Table S2. Western blotting analysis was performed as described previously^52^. Primary antibodies against the following proteins were used in this study: KRAS, RAS, phospho-MEK, phospho-ERK and β-actin (the antibodies against KRAS and β-actin were purchased from Santa Cruz Biotechnology Inc., and the other antibodies were purchased from Cell Signaling Technology).

### RNA *in situ* hybridization

MALAT1 was detected by *in situ* hybridization using a DIG-labelled probe, according to the manufacturer’s instructions (DIG application for ISH, ROCHE), and miR-217 using a double fluorescein –labelled probe (Exqion). Briefly, human tissue samples were fixed, embedded in optimal cutting temperature compound (OCT), and sectioned (at a thickness of 10 µm). The sections or PDAC cells were then treated with proteinase K (10 min in 2 mg/ml protease K) before being washed in DEPC-PBS and treated with RNase-free triethanolamine. Hybridization with 100 ng/ml digoxigenin (DIG)-labelled RNA probe or 20 nM fluorescein –labelled probe was performed overnight at 50 °C after prehybridization. For fluorescein –labelled probe, cells were sealed with DAPI. Images were acquired by fluorescence microscopy (Olympus) and overlapped using Image-Pro Plus (Version 6.0.0.260; Media Cybernetics, Inc., Tokyo, Japan). For the DIG-labelled probe, the sections were subsequently incubated with a 1:5000 dilution of anti-DIG-AP (alkaline phosphatase-conjugated; Roche), and 5-bromo-4-chloro-3-indolylphosphate/Nitro-blue tetrazolium was used for the colour reaction. The colour reaction produced a blue precipitate at the hybridization site. MALAT1 positivity was scored as negative (−), weakly or focally positive (1+), or strongly positive (2+).

### RNA and DNA transfection

We used two siRNAs to target MALAT1, one control oligonucleotide (GenePharma) and one miR-217 RNA mimic. Their sequences are listed in Supplementary Table S2. The siRNAs were transfected into Panc-1 and Aspc-1 cells using Lipofectamine RNAimax (Invitrogen), and the DNA vectors were transfected (2 μg/ml) into PDAC cells using Lipofectamine 2000 (Invitrogen). Stable MALAT1-knockdown cells were transfected with the indicated shRNA (shMALAT1) and selected with 400 µg/ml G418/Geneticin. Has-miR-217 mimic or inhibitor oligonucleotides (50 nmol/l) were transfected into Panc-1 and Aspc-1 cells using Lipofectamine RNAimax (Invitrogen). For the miRNA and DNA vector combination experiments, PDAC cells were cotransfected with different combinations of 2 μg/ml DNA construct and oligonucleotides.

### Proliferation, apoptosis and cell cycle assays

Cell Counting Kit-8 (CCK-8) assay. The cells were plated in 96-well plates (2000 cells per well) and transfected with siRNAs within 24 hours. Lipofectamine RNAimax (Invitrogen) was used for siRNA and RNA mimic transfection. The medium was replaced with 100 µl of complete medium containing 10 µl of CCK-8 reagent at the indicated time, after which the cells incubated in a cell incubator for 1 hour at 37 °C. The absorbances were subsequently measured at 450 nm and 630 nm with a Vmax microplated spectrophotometer (Molecular Devices).

EdU (5-ethynyl-2′-deoxyuridine) assay. Cells were incubated with 50 μM EdU (Ribobio, Guangzhou, China) for 2 h at 37 °C, fixed with 4% formaldehyde, then stained with Apollo reaction cocktail and Hoechst 33342, protected from light. Images were acquired by fluorescence microscopy and overlapped using Image-Pro Plus (Version 6.0.0.260; Media Cybernetics, Inc., Tokyo, Japan). Anchorage-independent colony formation assay. A total of 1.5 ml of DMEM containing 10% foetal bovine serum and 0.5% agar was plated on the bottoms of six-well plates and stored at 4 °C to allow the agar to solidify. Then, 1.5 ml of cell suspension containing 1,000 cells in DMEM supplemented with 10% foetal bovine serum (FBS) and 0.35% agar was added to the base layer of the agar. Colonies were allowed to grow in a cell incubator for 21 days at 37 °C with 5% CO_2_ before imaging was performed.

#### Cell apoptosis

Cell apoptosis assay was performed on PDAC cells 48 hours after the cells were transfection with either siCT or siMALAT1-1/2 using a BD Accuri C6 Flow Cytometer (with Cflow Plus software) and an Annexin V-FITC Apoptosis Detection Kit I (BD Biosciences), in accordance with the manufacturer’s instructions.

#### Cell cycle analysis

Cell cycle analysis was performed on the PDAC cell lines 48 hours after transfection. The cells were collected after being washed twice with PBS and fixed in cold 70% ethanol for 6 hours at 4 °C. Then, the fixed cells were incubated with RNase A at 37 °C, stained with propidium iodide (PI), and assessed using a FACScan flow cytometer with MODFIT software (BD Biosciences).

#### Scratch/wound healing assay

A total of 2 × 10^5^ cells suspended in 1 ml of DMEM were seeded in 12-well chambers. An artificial wound was introduced in the cell monolayer 24 hours after transfection using a 10-µl pipette tip. The cells were washed three times with PBS, and the wells were refilled with FBS-free medium to inhibit cell proliferation. Photographs were taken at 0, 24 and 48 hours after wounding using an Eclipse Ti inverted microscope (Nikon), and image analysis was performed using Image-Pro Plus 6 software (Media Cybernetics).

#### Transwell invasion assay

Twenty-four hours after transfection, the PDAC cells suspended in the medium without serum were seeded in cell culture inserts (Corning) coated with Basement Membrane Matrix (BD Biosciences). Then, the inserts were placed in a 24-well plate whose wells were each filled with 600 μl of complete medium containing 10% FBS, after which the cells were incubated for 48 hours. The noninvading cells were subsequently removed from the inserts, and the invasive cells located on the lower surface of the inserts were stained with haematoxylin and counted.

### Pancreatic tumour xenograft model

Five-week-old male BALB/c nude mice were used to examine the tumourigenicity of PANC-1 cells stably transfected with either a control hairpin shRNA (NC) or an shRNA targeting MALAT1. A total of 1 × 10^7^ cells suspended in PBS were subcutaneously inoculated into the dorsal flanks of 10 mice (5 mice received NC-shRNA cells, and 5 mice received MALAT1-shRNA cells). These mice were monitored throughout the experiments for inoculation-induced complications. Tumour size was measured twice weekly for one month using a calliper, and tumour volumes were calculated using the following formula: *V* = width^2^ × length/2 (mm^3^). At the end of the experiments, the mice were anesthetized with isoflurane and euthanized by cervical dislocation. Then, the tumours were removed, weighed and frozen in liquid nitrogen. This study was approved by the Animal Experiments Committee of the Chinese Academy of Medical Sciences and Peking Union Medical College.

### Dual-luciferase reporter assay

PDAC cells were cotransfected with 50 ng of pmirGLO-MALAT1-WT vectors or pmirGLO-MALAT1-MUT vectors along with miR-217 or negative control RNA, and pRL-TK-KRAS-WT/MUT vectors were cotransfected with pGL3 control plasmids to monitor transfection efficiency. Luciferase reporter assay was performed 24 hours after transfection using a dual-luciferase reporter assay system (Promega Corporation), according to the manufacturer’s instructions.

### Statistical analysis

Statistical comparisons between different groups were performed using Student’s t-test. Kaplan-Meier analysis and the log-rank test were applied to evaluate the prognostic significance of gene expression levels with respect to patient survival. A p-value < 0.05 was considered statistically significant. All statistical analyses were performed using SPSS version 17.0 software.

## Electronic supplementary material

supplement information
