# Supplementary material for: The lncRNA MALAT1 acts as a competing endogenous RNA to regulate KRAS expression by sponging miR-217 in pancreatic ductal adenocarcinoma
Source: Sci Rep. 2017 Jul 12;7:5186. doi: 10.1038/s41598-017-05274-4 (PMC5507931; doi:10.1038/s41598-017-05274-4)
Supplement: Supplementary file 1 — supplement information [file 41598_2017_5274_MOESM1_ESM.pdf]

The lncRNA MALAT1 acts as a competing endogenous RNA to regulate KRAS exprssion by sponging miR-217 in pancreatic ductal adenocarcinoma

Pingping Liu[1] ,Haiyan Yang[1], Jing Zhang[2], Xiaozhong Peng[3], Zhaohui Lu[1],Weimin Tong[3], Jie Chen[1,]*

[1] Department of Pathology, Peking Union Medical College Hospital, Chinese Academy of Medical Sciences and Peking Union Medical College, Beijing, 100730, China
[2] The State Key Laboratory of Medical Molecular Biology and [3] Department of Pathology, Institute of Basic Medical Sciences and School of Basic Medicine, Chinese Academy of Medical Sciences and Peking Union Medical College, Beijing, 100005, China

* To whom correspondence should be addressed. Tel: + 86-10-69159362; Fax: +86-10-69159362; Email: xhblk@163.com

Fig.S1

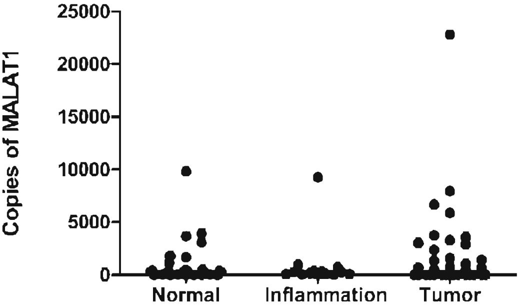

25000
20000
15000
10000
5000
0
Copies of MALAT1
Normal
Inflammation
Tumor

Fig.S2

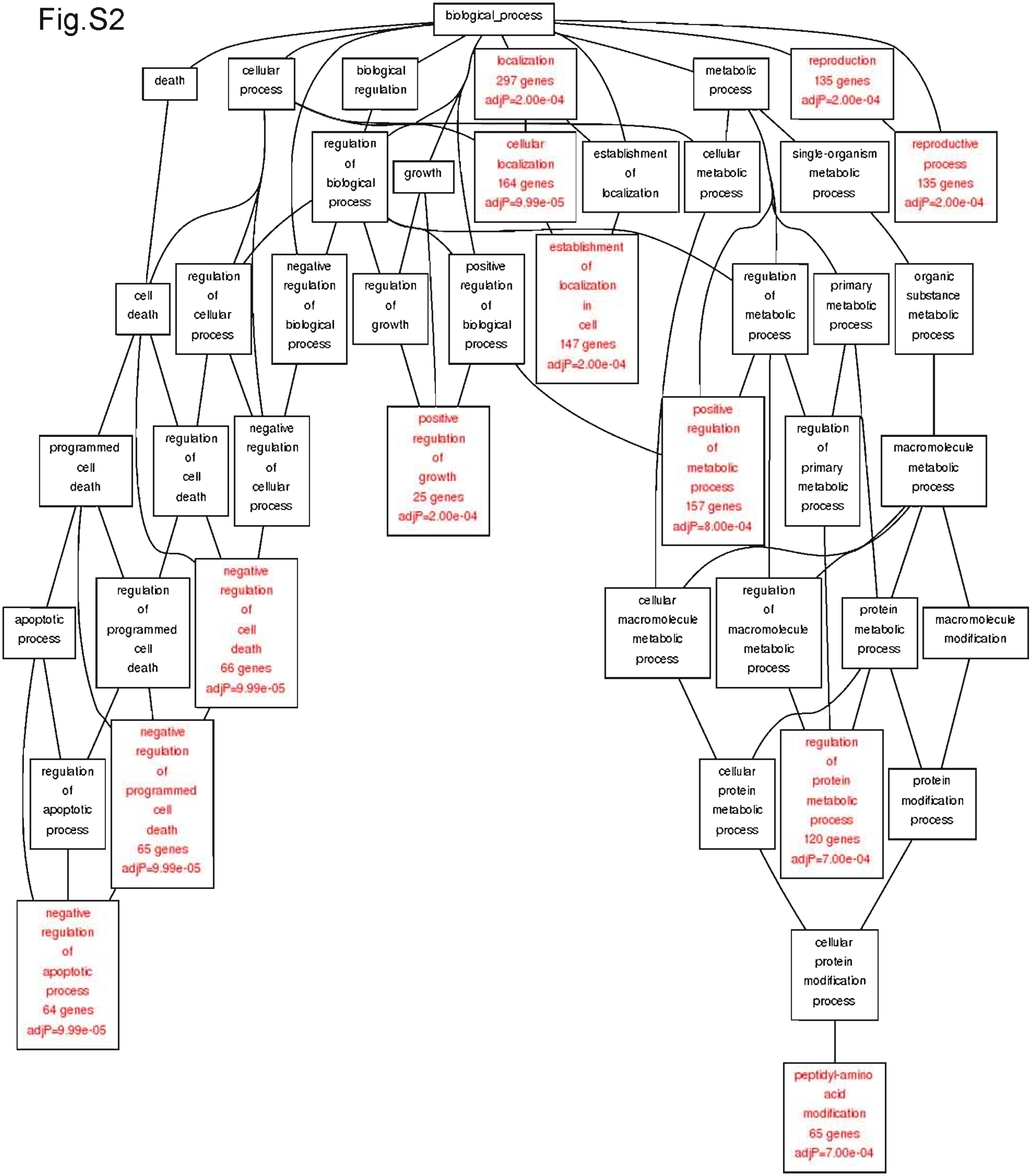

biological_process
death
cellular process
biological regulation
localization 297 genes adjP=2.00e-04
metabolic process
reproduction 135 genes adjP=2.00e-04
regulation of biological process
growth
cellular localization 164 genes adjP=9.99e-05
establishment of localization
cellular metabolic process
single-organism metabolic process
reproductive process 135 genes adjP=2.00e-04
cell death
regulation of cellular process
negative regulation of biological process
regulation of growth
positive regulation of biological process
establishment of localization in cell 147 genes adjP=2.00e-04
regulation of metabolic process
primary metabolic process
organic substance metabolic process
programmed cell death
regulation of cell death
negative regulation of cellular process
positive regulation of growth 25 genes adjP=2.00e-04
positive regulation of metabolic process 157 genes adjP=8.00e-04
regulation of primary metabolic process
macromolecule metabolic process
apoptotic process
regulation of programmed cell death
negative regulation of cell death 66 genes adjP=9.99e-05
cellular macromolecule metabolic process
regulation of macromolecule metabolic process
protein metabolic process
macromolecule modification
regulation of apoptotic process
negative regulation of programmed cell death 65 genes adjP=9.99e-05
cellular protein metabolic process
regulation of protein metabolic process 120 genes adjP=7.00e-04
protein modification process
negative regulation of apoptotic process 64 genes adjP=9.99e-05
cellular protein modification process
peptidyl-amino acid modification 65 genes adjP=7.00e-04

Fig.S3

1.WT: 5′…TAAGTTTAACTTGCATCTGCAGTATTGCATGTTAGGGATAAGTGCTT…3′
MUT: 5′…TAAGTTTAACTTGCATCAATTCCATTGCATGTTAGGGATAAGTGCTT…3′

2.WT: 5′…TTTTTTTTTTTTACAGACTTCACAGAGAATGCAGTTGTCTTGACTTC…3′
MUT: 5′…TTTTTTTTTTTTACAGACTTCACAGAGAGGAACCATGTCTTGACTTC…3′

3.WT: 5′…CTGTCTGTTCTGTTGGCAAGTAAATGCAGTACTGTTCTGATCCCGC…3′
MUT: 5′…CTGTCTGTTCTGTTGGCAAGTAAGGCCTTAACTGTTCTGATCCCGC…3′

Fig.S4

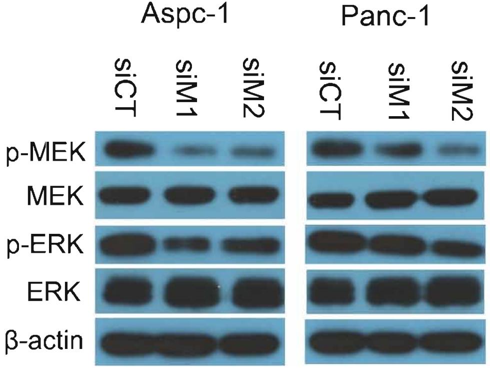
Aspc-1
Panc-1
siCT
siM1
siM2
siCT
siM1
siM2
p-MEK
MEK
p-ERK
ERK
β-actin

Fig.S5

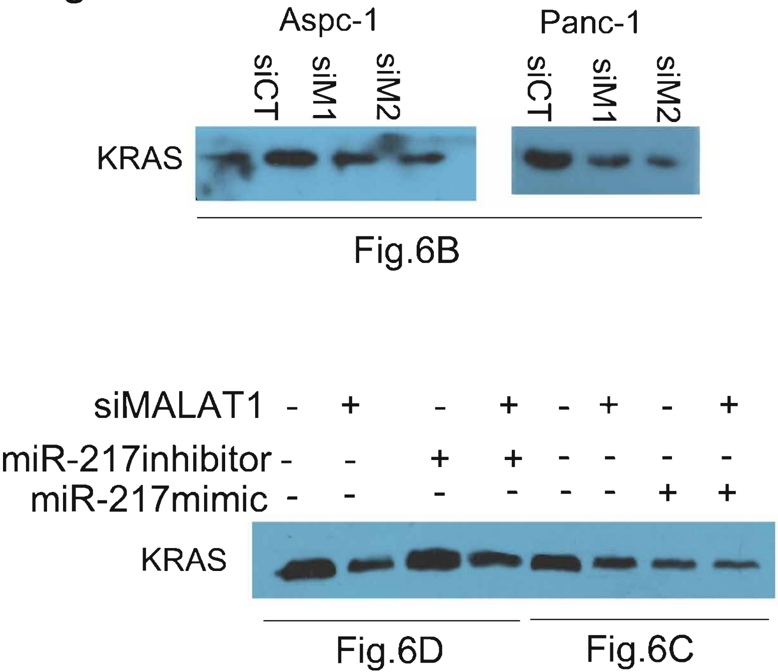
Aspc-1
Panc-1
siCT
siM1
siM2
siCT
siM1
siM2
KRAS
Fig.6B
siMALAT1 - + - + - + - +
miR-217inhibitor - - + + - - - -
miR-217mimic - - - - - - + +
KRAS
Fig.6D
Fig.6C

Table S1

		OS time	OS event	T stage	N stage	Biomaterial provider
GSM536454	PH06-12553T	17	1	2	1	Johns Hopkins Medical Institutions
GSM536455	PH10162T	2	1	3	1	Johns Hopkins Medical Institutions
GSM536456	PH11632T	14	1	3	1	Johns Hopkins Medical Institutions
GSM536457	PH15861T	4	1	3	1	Johns Hopkins Medical Institutions
GSM536458	PH16649T	1	0	3	1	Johns Hopkins Medical Institutions
GSM536459	PH17733T	29	0	3	1	Johns Hopkins Medical Institutions
GSM536460	PH17803T	1	0	3	1	Johns Hopkins Medical Institutions
GSM536461	PH19267T	8	1	3	1	Johns Hopkins Medical Institutions
GSM536462	PH19682T	54	1	3	1	Johns Hopkins Medical Institutions
GSM536463	PH19770T	6	1	3	1	Johns Hopkins Medical Institutions
GSM536464	PH20548T	7	1	3	1	Johns Hopkins Medical Institutions
GSM536465	PH20852T	7	1	2	1	Johns Hopkins Medical Institutions
GSM536466	PH21887T	13	1	3	1	Johns Hopkins Medical Institutions
GSM536467	PH21935T	16	1	3	1	Johns Hopkins Medical Institutions
GSM536468	PH22143T	15	1	3	1	Johns Hopkins Medical Institutions
GSM536469	PH22420T	6	1	4	1	Johns Hopkins Medical Institutions
GSM536470	PH23822T	7	1	3	1	Johns Hopkins Medical Institutions
GSM536471	PH25396T	18	1	3	1	Johns Hopkins Medical Institutions
GSM536472	PH27238T	7	1	2	0	Johns Hopkins Medical Institutions
GSM536473	PH3032T	22	1	3	0	Johns Hopkins Medical Institutions
GSM536474	PH32757T	25	1	2	1	Johns Hopkins Medical Institutions
GSM536475	PH33566T	30	0	3	1	Johns Hopkins Medical Institutions
GSM536476	PH35343T	22	1	3	1	Johns Hopkins Medical Institutions
GSM536477	PH35398T	6	1	2	1	Johns Hopkins Medical Institutions
GSM536478	PH35636T	14	1	3	1	Johns Hopkins Medical Institutions
GSM536479	PH36746T	1	0	3	1	Johns Hopkins Medical Institutions
GSM536480	PH37202T	6	0	3	1	Johns Hopkins Medical Institutions
GSM536481	PH376T	7	1	2	1	Johns Hopkins Medical Institutions
GSM536482	PH3939T	19	1	3	1	Johns Hopkins Medical Institutions
GSM536483	PH39655T	3	1	3	1	Johns Hopkins Medical Institutions
GSM536484	PH41130T	19	1	3	1	Johns Hopkins Medical Institutions
GSM536485	PH42166T	17	1	3	1	Johns Hopkins Medical Institutions
GSM536486	PH765T	1	0	3	1	Johns Hopkins Medical Institutions
GSM536487	PH8942T	20	1	3	1	Johns Hopkins Medical Institutions
GSM536488	P271N Replicate	11	1	3	1	Northwestern Memorial Hospital
GSM536489	P109T	49	1	2	0	Northwestern Memorial Hospital
GSM536490	P110T	52	0	3	0	Northwestern Memorial Hospital
GSM536491	P112T	14	1	3	1	Northwestern Memorial Hospital
GSM536492	P120T	55	0	2	0	Northwestern Memorial Hospital
GSM536882	P122T	8	1	2	1	Northwestern Memorial Hospital
GSM536883	P124T	19	1	3	1	Northwestern Memorial Hospital
GSM536884	P128T	52	0	3	0	Northwestern Memorial Hospital
GSM536885	P130T	18	1	3	0	Northwestern Memorial Hospital

GSM536886	P131T	29	1	3	1	Northwestern Memorial Hospital
GSM536887	P138T	43	0	1	0	Northwestern Memorial Hospital
GSM536888	P139T	6	1	3	1	Northwestern Memorial Hospital
GSM536889	P146T	18	1	3	0	Northwestern Memorial Hospital
GSM536890	P147T	42	0	3	0	Northwestern Memorial Hospital
GSM536891	P148T	46	0	3	0	Northwestern Memorial Hospital
GSM536892	P152T	20	1	NA	0	Northwestern Memorial Hospital
GSM536893	P162T	35	1	3	1	Northwestern Memorial Hospital
GSM536894	P164T redo	13	1	3	1	Northwestern Memorial Hospital
GSM536895	P174T	21	1	3	1	Northwestern Memorial Hospital
GSM536896	P178T	30	0	3	1	Northwestern Memorial Hospital
GSM536897	P180T Replicate	33	0	3	1	Northwestern Memorial Hospital
GSM536898	P182T	31	1	2	1	Northwestern Memorial Hospital
GSM536899	P183T	35	0	3	0	Northwestern Memorial Hospital
GSM536900	P184T	24	0	3	1	Northwestern Memorial Hospital
GSM536901	P188T IPMN	6	1	1	1	Northwestern Memorial Hospital
GSM536902	P190T	13	1	3	1	Northwestern Memorial Hospital
GSM536903	P194T	8	1	3	0	Northwestern Memorial Hospital
GSM536904	P214T	18	0	2	0	Northwestern Memorial Hospital
GSM536905	P227T	4	1	3	1	Northwestern Memorial Hospital
GSM536906	P235T	22	0	3	0	Northwestern Memorial Hospital
GSM536907	P237T	6	1	3	0	Northwestern Memorial Hospital
GSM536908	P241T	10	1	3	1	Northwestern Memorial Hospital
GSM536909	P242T	25	0	3	1	Northwestern Memorial Hospital
GSM536910	P245T	18	1	3	0	Northwestern Memorial Hospital
GSM536911	P248T	13	1	3	1	Northwestern Memorial Hospital
GSM536917	P249T	19	0	3	1	Northwestern Memorial Hospital
GSM536918	P258T	21	0	3	0	Northwestern Memorial Hospital
GSM536919	P260T	23	0	3	1	Northwestern Memorial Hospital
GSM536920	P268T	15	1	3	1	Northwestern Memorial Hospital
GSM536921	P272T	4	1	3	0	Northwestern Memorial Hospital
GSM536922	P275T Replicate	13	0	3	0	Northwestern Memorial Hospital
GSM536923	P31T Replicate	1	1	3	0	Northwestern Memorial Hospital
GSM536924	P35T	10	1		1	Northwestern Memorial Hospital
GSM536925	P40T	59	0	3	1	Northwestern Memorial Hospital
GSM536926	P41T Replicate	21	1	3	1	Northwestern Memorial Hospital
GSM536927	P42T	41	1		0	Northwestern Memorial Hospital
GSM536928	P63T	23	1	2	0	Northwestern Memorial Hospital
GSM536929	P85T	7	1	3	0	Northwestern Memorial Hospital
GSM536930	P92T	18	1	3	1	Northwestern Memorial Hospital
GSM536931		7	1	3	1	NorthShore University HealthSystem
GSM536932	PE182T	3	1	3	1	NorthShore University HealthSystem
GSM536933	E215T	3	1	2	1	NorthShore University HealthSystem
GSM536934	PE299T	15	1	3	1	NorthShore University HealthSystem
GSM536935	PE319T	26	1	3	0	NorthShore University HealthSystem

GSM536936	PE323T	13	1	2	0	NorthShore University HealthSystem
GSM536937	PE402T	33	0	3	1	NorthShore University HealthSystem
GSM536938	PE545T	8	1	3	0	NorthShore University HealthSystem
GSM536939	PE548T	4	1	3	1	NorthShore University HealthSystem
GSM536940	PE621T	17	0	2		NorthShore University HealthSystem
GSM536941	PE701T	15	0	3	1	NorthShore University HealthSystem
GSM536942	PE704T	13	0	3	1	NorthShore University HealthSystem
GSM536943	PE715T	4	0	3	1	NorthShore University HealthSystem
GSM536944	PE716T	10	0	3	1	NorthShore University HealthSystem
GSM536945	PE718T	2	0	3	1	NorthShore University HealthSystem
GSM536946	PE734T	8	0		1	NorthShore University HealthSystem
GSM536947	PE741T	7	0	3	1	NorthShore University HealthSystem
GSM536948	PE751T	8	0	3	1	NorthShore University HealthSystem
GSM536949	PE763T	8	0	2	1	NorthShore University HealthSystem

Table S2

Name	Sequences	Use
MALAT1	F: AAAGCAAGGTCTCCCCACAAG	qPCR
	R: GGTCTGTGCTAGATCAAAAGGCA	
GAPDH	F: GCACCGTCAAGGCTGAGAAC	qPCR
	R:GCCTTCTCCATGGTGGTGAA	
KRAS	F:ATTGTGAATGTTGGTGT	qPCR
	R:GAAGGTCTCAACTGAAATT	
XIST	F:GGATGTCAAAAGATCGGCCC	qPCR
	R:GTCCTCAGGTCTCACATGCT	
miR-217	GTCGTATCCAGTGCAGGGTCCGAGGTATTCGCACTGGATACGACtccaat	RT
miR-217	F:CGCTCtactgcatcaggaactga	qPCR
	R: GTGCAGGGTCCGAGGT	
U6	5'-CGCTTCACGAATTTGCGTGTCAT-3'	RT
U6	F:GCTTCGGCAGCACATATACTAAAAT	qPCR
	R:CGCTTCACGAATTTGCGTGTCAT	
MD-miniRNA	F:CTTCCCTAGGGGATTTCAGG	qPCR
	R:GCCCACAGGAACAAGTCCTA	
MALAT1-HindIII-F	5'-CCCAAGCTTTCCTAAAGGCAAATGACTCAA -3'	DNA constsruct for Northern blot
MALAT1-EcoRI-R	5'-CCGGAATTCAAAGAGAACCACACACTACC -3'	
Negative control	5'-GUACCUGACUAGUCGCAGATT-3'	Control of siRNA
siMALAT1-1	5'-GAUCCAUAAUCGGUUUCAATT-3'	Knock down of MALAT1
siMALAT1-2	5'-CACAGGGAAAGCGAGUGGUUGGUAATT-3'	Knock down of MALAT1
miR-217 mimics	5'-UACUGCAUCAGGAACUGAUUGGA-3'	overexpression of has-miR-217
miR-217 inhibitors	UCCAAUCAGUUCCUGAUGCAGUA	Knock down of has-miR-217

Titles and legends to figures and tables.

Fig. S1. Expression of MALAT1 fragments in human serum of patients with PDAC or chronic pancreatitis and healthy controls.

Fig. S2. Gene ontology analysis using the gene profiling results (GSE21051), showing the gene set differences between patients with high and low MALAT1 expression.

Fig. S3. Wild-type (WT) and three mutated (MUT) seed recognizing sites were subcloned into a pmirGLO luciferase reporter construct. The WT seed recognizing sites are shown in red, and the bases substituted in MUT are underlined.

Fig. S4. MAPK pathway protein expression in pancreatic cancer cells after transfection with negative control siRNA (siCT) or siMALAT1-1, either with or without an miR-217 mimic or an miR-217 inhibitor.

Fig. S5. The full-length gels and blots for KRAS protein.

Table S1. Survival and clinical data for patients with pancreatic cancer.

Table S2. Primers used for qPCR and RT, and sequences for siRNAs.
